# Visualization of *in vivo* protein–protein interactions in plants

**DOI:** 10.1093/jxb/erac139

**Published:** 2022-04-08

**Authors:** Vivien I Strotmann, Yvonne Stahl

**Affiliations:** Institute for Developmental Genetics, Heinrich-Heine University, Universitätsstr. 1, D-40225 Düsseldorf, Germany; Institute for Developmental Genetics, Heinrich-Heine University, Universitätsstr. 1, D-40225 Düsseldorf, Germany; MPI for Molecular Plant Physiology, Germany

**Keywords:** BiFC, FRET, FRET-APB, FRET-FLIM, *in planta*, *in vivo*, protein–protein interaction (PPI), splitLuc

## Abstract

Molecular processes depend on the concerted and dynamic interactions of proteins, either by one-on-one interactions of the same or different proteins or by the assembly of larger protein complexes consisting of many different proteins. Here, not only the protein–protein interaction (PPI) itself, but also the localization and activity of the protein of interest (POI) within the cell is essential. Therefore, in all cell biological experiments, preserving the spatio-temporal state of one POI relative to another is key to understanding the underlying complex and dynamic regulatory mechanisms *in vivo*. In this review, we examine some of the applicable techniques to measure PPIs *in planta* as well as recent combinatorial advances of PPI methods to measure the formation of higher order complexes with an emphasis on *in vivo* imaging techniques. We compare the different methods and discuss their benefits and potential pitfalls to facilitate the selection of appropriate techniques by providing a comprehensive overview of how to measure *in vivo* PPIs in plants.

## Teaser

This review aims to provide a comprehensive and critical overview of techniques used to measure protein–protein interactions in living plants.

## Introduction

*In vivo* protein–protein interaction (PPI) measurements are needed to understand the dynamic and complex interactions of proteins underlying a plethora of biological processes in all living organisms. The observed PPI can also help to decipher the function of the involved proteins of interest (POIs), bringing them into a wider biological context. In this review, we focus on several different techniques that are available for PPI measurements *in planta*, either in heterologous systems or in stably transformed plants.

Independent of the technique used to measure PPIs, several important prerequisites must be met for PPIs of two or more POIs to occur. Here, the most important prerequisite of PPI is the spatio-temporal co-localization of POIs at a subcellular level, for example at the plasma membrane in a certain tissue at a specific time during plant development. Depending on the plant species under investigation, information about a specific POI, for example gene expression, protein localization, and putative interaction domains, might already be available and can be utilized to design subsequent PPI experiments.

To determine whether two or more proteins are co-localized, the proteins can be visualized using different techniques such as immunolocalization with specific, fluorescent dye-labelled antibodies against the POI in fixed samples, or in living cells using genetically encoded fluorescent proteins (FPs) fused to the POI. Subsequently, (co-)localization can be assessed via fluorescence microscopy. For example, the most commonly used FP, enhanced green fluorescent protein (eGFP), is a 28 kDa protein that forms a cylinder-like structure 4.2 nm long and 2.4 nm wide (Hink *et al*., 2000). Therefore, even when using state-of-the-art fluorescence microscopes, a mere co-localization of two POIs is no proof that they interact physically, as the lateral diffraction limit of light means the maximum resolution attainable by light microscopy is only ~250 nm. Even when super-resolution microscopy methods are applied with resolutions in the range of ~30 nm, there is still uncertainty as to whether a PPI takes place between the POIs (Won, 2009). Therefore, a number of different techniques to test for direct PPI *in vitro* and *in vivo* have been developed (Struk *et al.*, 2019). In this review, we will focus on some of the traditional and newly emerging PPI techniques, with a focus on *in vivo* imaging techniques *in planta*.

Traditionally, techniques such as co-immunoprecipitation (co-IP) or yeast two-hybrid (Y2H) experiments were used to measure PPI. These techniques allow for the identification of numerous potential interaction candidates in a short time and are thus regarded as relatively high throughput. Although the use of these methods results in the loss of spatial and temporal information about the POIs and their interaction partners, they can still serve as a starting point to identify potential interacting POIs, which can then be verified by subsequent *in vivo* PPI measurements.

## Traditional methods

### Co-immunoprecipitation

The *in vitro* method co-IP is still one of the most commonly used techniques to identify PPIs (Ren *et al.*, 2003; Phee *et al*., 2006). During co-IP, an immobilized antibody against the POI isolates the POI from a cell lysate, along with other proteins that directly or indirectly interact with the POI (Ransone, 1995; Ren *et al.*, 2003; Phee *et al.*, 2006). These potential interaction partners can then be identified by mass-spectroscopy (MS) (as reviewed in Monti *et al.*, 2005). Furthermore, the precipitated complex can be tested for a specific target protein that has been identified by other experiments. Additionally, the putative interaction partner can be subsequently confirmed and visualized by a western blot using an antibody against the identified complex partner.

However, this is also one of the major limitations of this technique, as protein-specific antibodies are, at least in plant biology, often not available. Therefore, it is necessary to label the POI with one of the common protein tags available (e.g. His, FLAG, HA, or FPs), which can then be detected. Another concern is that co-IP is not well suited to detect weak or transient interactions as the experimental procedure includes several washing steps, often with detergents, to eliminate non-specific binding. Also, detection of proteins via western blot requires sufficient protein expression, which can be problematic if endogenous promoters are used, which are often only weakly or transiently expressed, or only expressed in a few cells (as reviewed in Tang and Takahashi, 2018). Another major drawback of this technique is that spatial information of the POI is lost through lysis of the cells. Here, proteins that are not present in the same cellular compartments are released into the lysate and PPIs may take place, even if they would not normally come into contact with each other in an intact cell. Additionally, when using co-IP, it remains unclear whether the discovered interaction is direct or indirect (as reviewed in Masters, 2004). Therefore, false-positive results must be considered, and PPI must be confirmed using other techniques that conserve spatial information. Furthermore, false-negative results are also possible due to the need for the POI to be soluble, which, for example in the case of membrane proteins, is not always the case. Nevertheless, co-IP with subsequent MS offers the possibility to identify a multitude of novel interaction partners which can subsequently be confirmed using other techniques as described below.

### Yeast two-hybrid

A widely used high-throughput *in vivo* technique is the Y2H system, which was originally designed to identify PPIs using the yeast GAL4 transcriptional activator. GAL4 consists of two functional domains, a DNA-binding domain and an activator domain (Fields and Song, 1989). In the Y2H system, the binding of GAL4 to the upstream activation sequence triggers the transcription of an enzymatic reporter gene, for example *lacZ* coding for β-galactosidase. Separation of these two domains and fusion to two different POIs allows testing of their interaction with an easy read-out, such as a colour reaction triggered by the addition of a suitable substrate for β-galactosidase. In recent decades, several other variations of this technique have been developed, for example in *Arabidopsis thaliana* protoplasts, named protoplast two-hybrid (Ehlert *et al.*, 2006).

A Y2H screen can give an indication of whether a PPI could take place between two POIs, and it is easy to carry out without the need for any sophisticated equipment. In addition, Y2H offers the opportunity for large-scale high-throughput approaches and allows screening of thousands of proteins for potential PPIs, which has led to the availability of Y2H interactome databases (e.g. Arabidopsis Interactome Mapping Consortium, 2011 for *A. thaliana*). The analysis of such large networks of interacting proteins offers the opportunity to classify POIs into larger biological contexts and enables the discovery of novel hypothetical links and putative functions of POIs. Even though these databases, if available, are a very good starting point, one major limitation of the underlying Y2H technique is the high rate of false-positive, but also false-negative results. Here, estimates suggest 70% false-positive identifications, which are caused by the overexpression of the POI and the expression in a heterologous system (as reviewed in Deane *et al.*, 2002; Auerbach and Stagljar, 2005). Nevertheless, high-throughput Y2H screens are very useful to identify many putative POIs that could interact, even though other promising *in vivo* high-throughput methods have been developed recently.

### Biotin-based proximity labelling

Within the last few years, an enzyme-catalysed proximity labelling technique was developed in which biomolecules, usually proteins or RNA, are labelled if they are in close proximity to the POI (Roux *et al.*, 2012). Here, a POI is fused to a ligase that covalently labels adjacent proteins or RNA with biotin. The biotinylated proteins can then be isolated using streptavidin which has a strong affinity for biotin. The putatively interacting proteins pulled-down in this way can then be identified by MS. The bifunctional BirA isolated from *Escherichia coli* is one of the best studied biotin ligases (Chakravartty and Cronan, 2012). Due to its high sequence specificity, BirA was not only used for protein isolation employing streptavidin, but also to analyse binary interactions. Fusing two POIs with BirA or BAP, a short biotin acceptor peptide, respectively, leads to biotinylation in the case of interaction of the POIs (as reviewed in Kim and Roux, 2016). Since the discovery of this useful mechanism, many improvements of biotin ligases have been developed. BioID is a mutated biotin ligase derived from BirA and works independently of BAP, leading to unspecific labelling of all nearby biomolecules. Recently another modification of BirA led to the development of the biotin ligase TurboID (Branon *et al.*, 2018). It combines advantages of other commonly used proximity labelling enzymes, such as APEX2 and BioID, by enabling non-toxic, fast labelling and an increased catalytic efficiency.

To date, one of the major limitations of proximity labelling in plants has been that experiments had to be carried out at 37 °C due to the temperature requirements of the labelling enzyme, which could cause heat stress in plants. However, TurboID can be successfully used at room temperature in transiently and stably transformed *Nicotiana benthamiana* and stably transformed Arabidopsis, thereby avoiding unnecessary abiotic stress in the plants (Mair *et al.*, 2019; Zhang *et al.*, 2019). In contrast to co-IP approaches, proximity labelling is also suitable to detect low-affinity or transient interactions, due to the promiscuity of the biotin ligases and the strong biotin–streptavidin affinity (as reviewed in Kim and Roux, 2016). Additionally, the labelling of the putative interaction/complex partner occurs under native spatio-temporal conditions *in vivo*; only the subsequent identification takes place *ex vivo*. Nevertheless, some caveats must be considered even when TurboID or the smaller version, called miniTurbo, are used; for example, biotin might not be accessible to some organelles such as peroxisomes and vacuoles because of their acidic environments (Mair *et al.*, 2019). Furthermore, unspecific background labelling can result in false-positive potential complex/interaction partners, and therefore appropriate negative controls must be included. In addition, as when using co-IP to monitor PPIs, isolated interaction partners must be identified by MS. Lastly, it must be taken into account that the labelling process requires a negatively charged amino acid on the surface of the protein, which could lead to false-negative results if negatively charged amino acid are not available in some POIs (as reviewed in Yang *et al.*, 2021).

All of the three above-mentioned techniques can identify a multitude of different putatively interacting proteins. Nevertheless, these interactions must be verified individually by other PPI methods, and we will focus on these PPI methods in the following sections.

## Shedding light on *in vivo* PPI measurements

*In vivo* visualization and quantification of PPIs has greatly profited during the past decades from the use of luminescent proteins and, notably, FPs that emit photons in a specific spectral range. The high spatio-temporal resolution at which FP-tagged POIs can be detected *in vivo* without the need for further substrates or cofactors has led to the application of a multitude of different quantitative methods involving FPs *in planta* (Grossmann *et al.*, 2018). We will describe techniques that allow for *in vivo* PPI detection using fluorescence or bioluminescence and discuss potential benefits and pitfalls of the different applications. Next, we introduce two techniques to measure PPI by protein fragment complementation using either fluorescence or luminescence as a read-out.

### Bimolecular fluorescence complementation (BiFC)

**Fig. 1.**
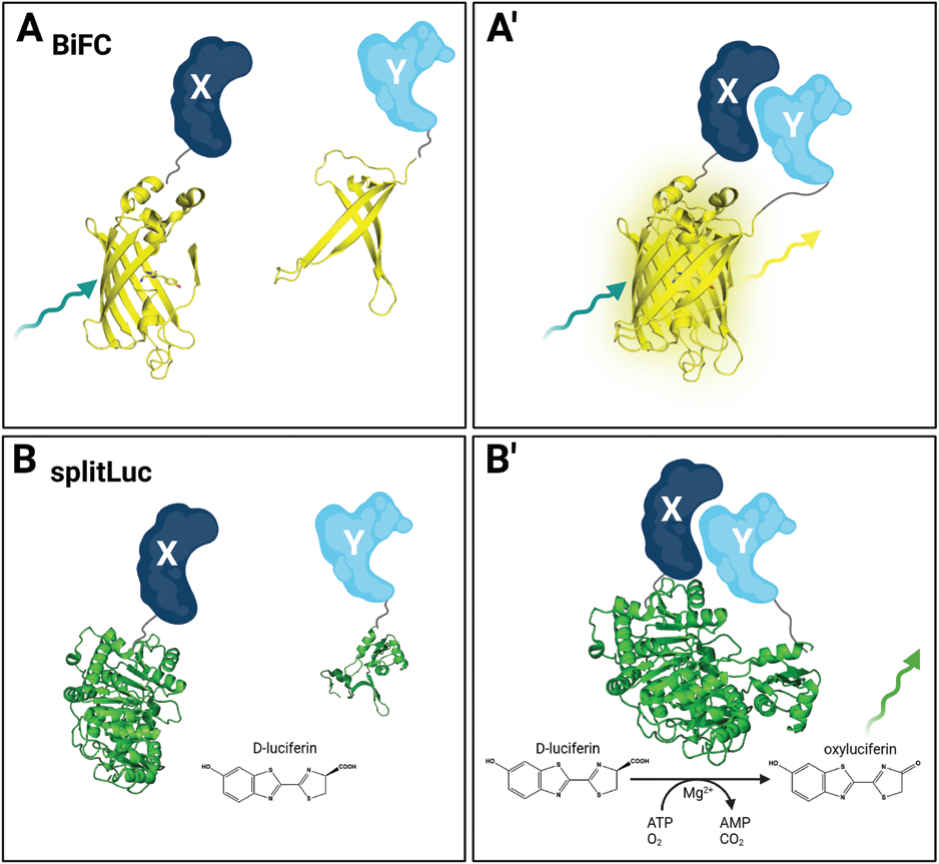
Split systems for measuring PPI *in vivo*. (A) In the bimolecular fluorescence complementation (BiFC) system, two POIs (X, dark blue; Y, light blue) are fused to the N- or C-terminal part of an FP (here, 3D structures of YFP in yellow), respectively. (Aʹ) If the two POIs interact, the two YFP parts are reconstituted and, after excitation (teal arrow), can emit light (yellow arrow). (B) In the split-luciferase (splitLuc) system, two POIs (X, dark blue; Y, light blue) are fused to the N- or C-terminal part of a luciferase (here, 3D protein structure of firefly luciferase in green). (Bʹ) If the two POIs interact, the luciferase fragments are reconstituted and can produce light (green arrow) in the presence of the substrate d-luciferin, in an ATP- and oxygen-dependent reaction. Figure created with BioRender.com.

The *in vivo* BiFC assay is based on the complementation of an FP (Hu *et al.*, 2002; Bracha-Drori *et al.*, 2004). Here, the two POIs are fused to the N- or C-terminal part of the FP, respectively. If the two POIs are in close proximity to each other, the FP is reconstituted and its fluorescence restored, thus indicating interaction of the POIs (see Fig. 1B, Bʹ). This technique was first described using yellow fluorescent protein (YFP), but since then other split FPs have become available (Hu *et al.*, 2002; Fan *et al.*, 2008). This opens up the possibility to simultaneously visualize several PPIs within the same cell, known as multicolour BiFC (Hu and Kerppola, 2003; Waadt *et al.*, 2008). The relatively simple principle of detecting interactions makes BiFC a widely used technique, since it does not require any specialized equipment other than a fluorescence microscope and appropriate filters for excitation and emission. Additionally, PPIs can be directly visualized in different cell compartments and, because of the cellular resolution of modern fluorescence microscopes or laser scanning microscopes, BiFC can provide information about the spatial characteristics of the investigated interaction. Because of its simplicity, many PPIs could be verified by BiFC *in vivo* (Table 2), for example in transient experiments in *N. benthamiana* and Arabidopsis leaf epidermal cells (Bracha-Drori *et al.*, 2004), mustard seedlings, and also in protoplasts of Arabidopsis (Olejnik *et al.*, 2011) and rice (Chen *et al.*, 2006).

**Table 2.** Examples of PPI measured *in planta*

Technique	Recent example and biological context	Organism	References
BiFC and FRET-FLIM	PII localizes to foci within chloroplasts where it interacts with NAGK and BCCP and thereby regulates protein degradation.	Transient expression in *N. benthamiana*	Krieger *et al.* (2021)
SplitLuc (firefly)	MKK2 and MPK2, both known to be involved in plant immunity, interact with ACO2 and with ACO2, CHH, and PBP1, respectively.	Transient expression in *N. benthamiana*	Leissing *et al*. (2021)
SplitLuc (firefly), BiFC, Y2H	OsUEV1B interaction with OsVDAC1 is suggested to be required for phosphate homeostasis in rice	Transient expression in *N. benthamiana* and Arabidopsis protoplasts	Liu *et al.* (2021)
FRET-APB	CRN and CLV2 interact at the plasma membrane, where they perceive CLV3 peptide and regulate stem cell number in the shoot apical meristem of *A. thaliana.*	Transient expression in *N. benthamiana*	Bleckmann *et al.* (2010)
FRET-FLIM	Cell type-specific interaction of the transcription factor network SHR–SCR–JKD regulates gene expression and thereby specifies cell fate in the *A. thaliana* root.	Stable expression in *A. thaliana*	Long *et al.* (2017)
FRET-FLIM, Y2H	OsFD7 involved in floral transition and panicle development in rice, was found to interact with OsFTL1, Hd3a, and RFT1.	Transient expression in onion peel cells	Kaur *et al.* (2021)
	BRAVO and WOX5 interaction is involved in quiescent centre quiescence. Both transcription factors can interact with the BES1–TPL repressor complex.	Transient expression in *N. benthamiana*	Betegón-Putze *et al.* (2021)
Triple FRET	BRI1 and BAK1 can form a trimeric complex with RLP44 which is thought to sense cell wall integrity in response to BR signalling.	Transient expression in *N. benthamiana*	Glöckner *et al.* (2020)
FRET-FLIM and homo-FRET	CLV1 and ACR4 form homo- and heteromeric complexes depending on their subcellular localization and thereby control distal root meristem maintenance in *A. thaliana*.	Transient expression in *N. benthamiana*	Stahl *et al.* (2013)
	Cluster formation of CRN, CLV2, and CLV1 at the plasma membrane in presence of CLV3 regulates shoot meristem maintenance in *A. thaliana*.	Transient expression in *N. benthamiana*	Somssich *et al.* (2015)
FCCS	Exogenous BR application leads to an increased co-localization of BRI1 and AtFlot1 and stimulation of the membrane microdomain-associated pathway of BRI1 internalization. Co-diffusion of BRI1 and CLC demonstrates that BRI1 internalization is clathrin dependent.	Stable expression in *A. thaliana*	Wang *et al*. (2015)
RICS and N&B	SHR was found to exist in a monomeric and dimeric state in the endodermis and both forms can interact with SCR, indicating a stoichiometric complex composition of 1:1 or 2:1.	Stable expression in *A. thaliana*	Clark *et al.* (2016)
KSP	Proof of concept as shown for the transcription factors: SACL and LHW; BES1 and BIN2 and the endocytosis protein complex: TPLATE and TML	Transient expression in *N. benthamiana*	Winkler *et al*. (2021)

Nevertheless, one major limitation of BiFC is the high frequency of false-positive PPIs caused by the intrinsic affinity of the two parts of the FP for each other (see Table 1) (as reviewed in Horstman *et al.*, 2014; Romei and Boxer, 2019). This is especially problematic when the expression is driven by a constitutive promoter in a heterologous system and thus the concentration of the proteins no longer reflects the endogenous expression level. In addition, expression in a heterologous system could also lead to false (co)-localization of proteins that, under native conditions, are localized in distinct compartments; for example, a protein expressed in the cytoplasm which is highly expressed in epidermal leaf cells of *N. benthamiana* could partially co-localize with a plasma membrane protein. Additionally, several attempts to diminish self-assembly, such as by changing the split position, have been made, but none of these approaches was generally applicable in plants (as reviewed in Horstman *et al.*, 2014).

**Table 1.** Evaluation of PPI techniques *in planta* involving imaging

Method	Cellular resolution	Dynamics	False positives	False negatives	Applicability	Special features and characteristics
BiFC	●	○	● ●	○ ●	● ●	Suitable for weak/transient PPIs
Split-Luc	○	●	●	●	●^* a*^	Dynamic assembly and disassembly of PPIs can be studied
FRET-APB	●	●	○ ●	●	● ●^* b*^	Fast data acquisition and analysis
FRET-FLIM	● ●	●	○ ●	●	○ ●^* c*^^.^^*d*^	High quality of acquired data
BiFC and FRET	● ●	○ ●	● ●	● ●	○ ●^* d*^^,^^*e*^	Analysis of trimeric complexes
Triple FRET	● ●	○ ●	○ ●	● ●	○ ●^* c*^^,^^*d*^^,^^*e*^	Analysis of trimeric complexes
Homo-FRET	● ●	● ●	○ ●	● ●	○ ●^* d*^	Analysis of higher order complexes Can be combined with FRET-FLIM
FCCS	○ ●	● ●	○	○ ●	○^*d*^	Low concentration of POI needed Simultaneous detection of PPI and dynamics
RICS and N&B	○ ●	● ●	○ ●	●	●^* c*^	Simultaneous detection of PPI and dynamics
KSP	○ ●	○ ●	○ ●	○ ●	○ ●^* f*^	Analysis of higher order complexes. Inducible visualization of PPI. Can be added to other methods as intrinsic positive control

‘○’ = ‘no or low’, ‘○ ●’ = ‘medium’, ‘●’ = ‘high’, ‘● ●’ = ‘very high’.

^*a*^ Phosphorescence of chlorophyll can mask the signal of luciferase.

^*b*^ Not suitable for moving proteins.

^*c*^ Data acquisition and analysis can be time consuming.

^*d*^ Special technical equipment might be needed,

^*e*^ Appropriate controls needed.

^*f*^ Rapamycin-induced effects must be considered.

Therefore, appropriate negative controls are absolutely necessary to minimize false-positive results (Horstman *et al.*, 2014). In addition, BiFC does not give any information about the temporal dynamics of PPI since the FP is highly unlikely to dissociate after reconstitution, as FPs have a half-life >24 h (Hu *et al.*, 2002). On the other hand, this phenomenon can also be advantageous in detecting weak and/or transient PPIs (Morell *et al.*, 2007). Recently, a split fluorescence reporter has been developed that also allows monitoring of assembly and disassembly of PPIs in living cells (Tebo and Gautier, 2019). To date, this reporter has only been applied in mammalian cell culture. Another *in vivo* protein fragment complementation assay based on a split-luciferase (splitLuc) can detect dynamic changes of PPI, and has been applied in plants; this is described next.

### Split-luciferase 

**Fig. 2.**
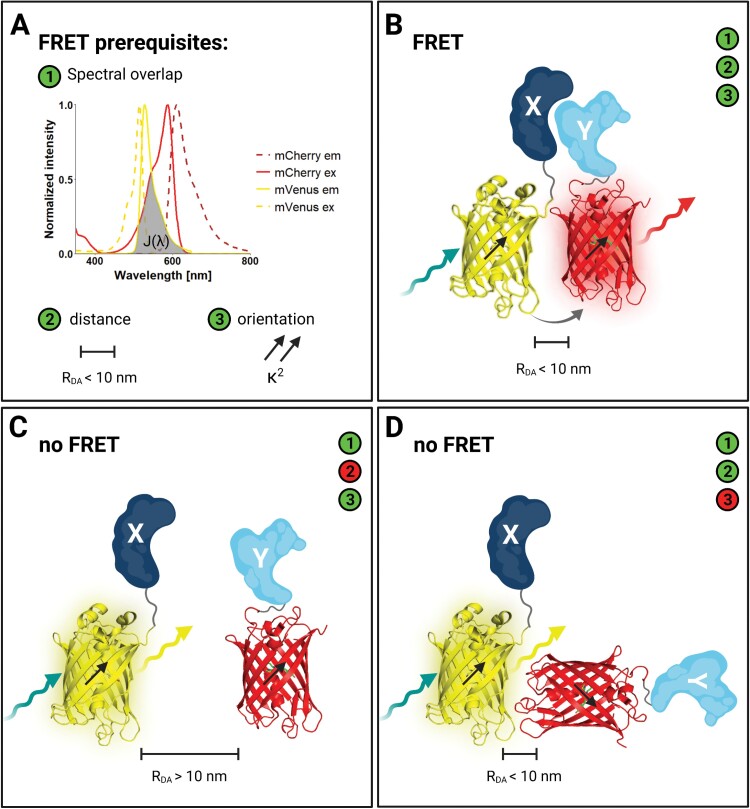
FRET prerequisites and possible scenarios when measuring PPI *in vivo.* (A) Prerequisites for FRET to take place between the two fluorophores or FPs of the chosen FRET pair, in this case the FPs mVenus (yellow) and mCherry (red), are: ① spectral overlap of donor emission and acceptor excitation [*J*(λ)]; ② distance between the donor and acceptor is <10 nm (R_DA_); ③ dipole orientation of the donor and acceptor are parallel (κ^2^). (B) PPI of two POIs can be measured by FRET *in vivo* if the donor FP (mVenus 3D structure in yellow) and the acceptor FP (mCherry 3D structure in red) are fused to POI X (dark blue) or Y (light blue), respectively. In the case that all three FRET prerequisites are met (①–③ green), FRET takes place by the energy migration after exciting (teal arrow) the donor FP to the acceptor FP (grey arrow) which is excited and can emit light (red arrow). (C) No FRET can be measured if the distance between donor and acceptor FPs (*R*_DA_) is >10 nm (red number ②). (D) No FRET can be measured if the dipole orientation (κ^2^) of donor and acceptor are not parallel (red number ③). Figure created with BioRender.com.

The *in vivo* splitLuc assay is based on the complementation of two fragments of a luciferase enzyme fused to two POIs that, in the case of interaction of the POIs, leads to enzyme reconstitution and subsequent substrate conversion. The turnover of the substrate, and therefore the interaction of the POIs, can be monitored by the emission of bioluminescence (Fig. 2A, Aʹ). *In planta*, two luciferases are most frequently used for splitLuc assays: one is obtained from the North American firefly (*Photinus pyralis*), referred to as firefly luciferase, and one from the sea pansy (*Renilla reniformis*), referred to as *Renilla* luciferase. The firefly luciferase uses d-luciferin as a substrate which is converted in a two-step ATP- and oxygen-dependent reaction to oxyluciferin, AMP, carbon dioxide, and light (de Wet *et al*., 1987). Interestingly, the emission spectrum of the firefly luciferase peaks at 560 nm (green light), but can undergo a red shift in an acidic environment or at higher temperatures (reviewed in Fraga, 2008). The *Renilla* luciferase converts its substrate coelenterazine in an oxygen-dependent reaction to coelenteramide, carbon dioxide, and blue light, with an emission maximum at 480 nm, and is ATP independent (Matthews *et al.*, 1977). Compared with the firefly luciferase with an approximate mol. wt of 62 kDa, the *Renilla* luciferase is relatively small, measuring 37 kDa (Matthews *et al.*, 1977; de Wet *et al*., 1987). The distinguishable emission spectra of these two different enzymes combined with a high substrate specificity allows for the simultaneous detection of the activity of both luciferases (McNabb *et al.*, 2005).

For both luciferases, a split variant is available for the detection of PPI (Ozawa *et al.*, 2001; Paulmurugan and Gambhir, 2003; Fujikawa and Kato, 2007; Chen *et al.*, 2008). These have been successfully utilized in different experimental approaches in plants, such as in transient expression in *N. benthamiana* (Liu *et al.*, 2021) or in Arabidopsis protoplasts (Li *et al.*, 2011) (see Table 2). Which luciferase is more suitable for the detection of PPI *in vivo* strongly depends on the characteristics of the putative interaction partners. For example, the enzymatic reaction catalysed by *Renilla* luciferase does not require ATP, which might be limiting in some cellular compartments. On the other hand, the substrate coelenterazine is unstable and can undergo spontaneous oxidation (Zhao *et al.*, 2004), whereas d-luciferin is more stable.

Compared with other protein fragment complementation assays, luciferase fragments do not spontaneously reconstitute, thus avoiding false-positive interactions often encountered in BiFC. Furthermore, splitLuc assays enable the investigation of dynamic changes of PPIs since the reconstitution of the firefly luciferase is reversible, as shown in large-scale approaches in Arabidopsis mesophyll protoplasts (Table 1) (Li *et al.*, 2011). The high turnover rate of reconstituted luciferase and its short half-life time allow the visualization of formation and dissociation of protein complexes (Luker *et al.*, 2004; Xing *et al.*, 2016). Although the splitLuc assays require the addition of a substrate, it can be easily applied exogenously, either by incubation or by watering (Chen *et al.*, 2008), or even through infiltration of the diluted substrate (Schatlowski *et al.*, 2010).

Despite its advantages, splitLuc assays are less commonly used than other protein fragment complementation assays. For one thing, luciferase activity can only be measured in the dark. Therefore, light-dependent processes are not suitable for this technique. Furthermore, for splitLuc assays in green tissue, for example tobacco leaves or Arabidopsis leaf protoplasts, a special filter is needed that excludes the phosphorescence of Chl *a* and *b* (reviewed in Krasnovsky and Kovalev, 2014) as this could interfere with the detection of the emitted light from the luciferase. Although splitLuc experiments can be used in quantitative high-throughput assays, such as on floating leaf discs or on protoplasts using plate readers (Chen *et al.*, 2006; Gehl *et al.*, 2011), these experiments do not provide information on the subcellular localization of the PPI and are not suitable for low-affinity interactions (reviewed in Xing *et al.*, 2016). Another concern is that compared with commonly used fluorescent proteins, such as GFP or mCherry, the firefly luciferase is particularly large, which could cause problems when monitoring interactions of small proteins as this could sterically hinder PPIs (Matthews *et al.*, 1977; de Wet *et al.*, 1987; Cormack *et al.*, 1996; Shaner *et al.*, 2004). Recently, a smaller so-called Nano luciferase (Nluc) with a total molecular mass of 19.1 kDa was established to address this concern (Wang *et al.*, 2020).

Nonetheless, neither BiFC nor splitLuc assays can provide information on whether the interaction of the proteins is direct or indirect or whether an interaction could be considered strong or weak, thereby making the quantification of PPI of different interaction partners impossible. Additionally, either spatial or temporal resolution of PPI is lost in splitLuc or BiFC experiments, respectively. Therefore, other quantitative techniques to measure *in vivo* PPI that preserve the spatio-temporal resolution are additionally required and will be discussed in the next sections.

## Measurement of PPI by energy transfer

### Förster resonance energy transfer (FRET)

FRET, first described by Theodor Förster (Förster, 1948), is a physical phenomenon in which the energy of an excited donor fluorophore is transferred by a radiation-free process to an acceptor chromophore, for example chlorophyll in the light-harvesting complexes necessary for plant photosynthesis. This process of energy transfer is strongly dependent on the distance between donor and acceptor fluorophores (*R*_DA_) (see Fig. 2A, C), the overlap integral of donor emission and acceptor absorption spectra [*J*(λ)] (see Fig. 2A), as well as the parallel orientation of the donor and acceptor dipoles (κ^2^) (see Fig. 2A, D). FRET efficiency (*E*_FRET_) is directly dependent on the distance between the donor and acceptor fluorophores, as it is inversely proportional to the sixth power of the distance between donor and acceptor, and can be described by the following equation:


$$\begin{array}{l} E_{FRET}=\;\;\;\frac{R_{0}^{6}}{R_{0}^{6\;\;\;}+\;\;\;R_{DA}^{6}} \end{array}$$

Here, *R*_DA_ represents the actual distance between the two fluorophores, and *R*_0_ the so-called Förster distance between the two fluorophores, a characteristic distance between a pair of fluorophores at which the FRET efficiency (*E*_FRET_) is 50%, which is usually well below 10 nm. For PPI measurements, two POIs are fused to suitable donor or acceptor fluorophores or FPs, also called FRET pairs. Widely used FP FRET pairs are: eCFP–eYFP, eGFP–mRFP, eGFP–mCherry, or mVenus–mCherry. The Förster radius (*R*_0_) of the FRET pair eCFP–eYFP is 4.9 nm (Bajar *et al.*, 2016). Because of its strong distance dependency, FRET can be utilized to quantitatively determine PPI *in vivo*. To figure out which FRET pair is most suitable to detect PPI in a specific *in planta* experiment can be quite challenging, as it depends on several aspects, such as spectral properties, photostability, folding, localization, and activity of the labelled POI within the respective cellular context (Denay *et al.*, 2019). The development of new FPs or new variants of already established FPs starts with analysing important characteristics *in vitro*, such as photostability, pH stability, and maturation time (reviewed in Day and Davidson, 2009). For some FPs, these attributes have been at least partially characterized *in vivo*, albeit mostly in bacteria (Megerle *et al.*, 2008; Hebisch *et al.*, 2013; Balleza *et al.*, 2018) and also in yeast or mammalian cell culture (Zhong *et al.*, 2019; Lee *et al.*, 2020). Therefore, when starting with a recently developed FP, its applicability in plants first needs to be tested, as its properties can vary quite significantly in comparison with published results from other organisms (Denay *et al.*, 2019).

Furthermore, when choosing a suitable FRET pair for PPI experiments, the maturation time of the individual fluorophores should also be considered. The specific maturation time of an FP can vary between different species or even within different strains of the same species, as shown for *E. coli* (Hebisch *et al.*, 2013). As higher amounts of acceptor increase the possibility for FRET to occur, an equal or shorter maturation time for the acceptor fluorophore compared with the donor fluorophore is preferred (Bajar *et al.*, 2016; Denay *et al.*, 2019).

One important property of FRET is that it also affects the mean fluorescence intensity and lifetime of the donor fluorophore, as the fraction of excited donor fluorophores is depopulated faster in the presence of a suitable acceptor fluorophore in close proximity (Bücherl *et al.*, 2014; Weidtkamp-Peters and Stahl, 2017). The resulting decrease of donor fluorescence intensity and lifetime, also called quenching, and the consequent increase of acceptor fluorescence can be measured by different approaches.

Donor and acceptor fluorescence intensities can be simultaneously measured by either acquiring a complete spectrum covering the donor and acceptor emission or by using appropriate filter sets for acceptor and donor fluorescence, known as sensitized emission. These techniques are often applied in genetically encoded FRET-based biosensors, detecting changes of intramolecular FRET in response to specific biological stimuli, such as calcium, reactive oxygen species, pH, phytohormones, and nutrients (Walia *et al.*, 2018).

The two techniques most widely used for quantitative measurements of PPI by FRET are acceptor photobleaching (APB) and fluorescence lifetime imaging microscopy (FLIM), which we describe in more detail below.

### Acceptor photobleaching (APB)

One of the most accessible FRET-based techniques to monitor PPI *in vivo* is acceptor APB. APB makes use of the differences in fluorescence intensity of the donor molecule in the presence or absence of the acceptor. Here, if FRET takes place, the energy transfer from the donor to the acceptor fluorophore is inhibited by bleaching the acceptor with a strong laser pulse (Bastiaens and Jovin, 1996; Bastiaens *et al.*, 1996; Wouters *et al.*, 1998; Kenworthy and Edidin, 1999; Kenworthy, 2001; Karpova *et al.*, 2003; Albertazzi *et al.*, 2009). Therefore, if the two POIs interact, bleaching of the acceptor leads to an increase of the donor fluorescence intensity because the energy is no longer transferred to the acceptor. This technique has successfully been applied *in planta*, for example in transiently expressing *N. benthamiana* leaf epidermal cells expressing different receptor proteins (Bleckmann *et al.*, 2010). An apparent FRET efficiency (*E*_FRET_, as a percentage) can be calculated if the intensity of the donor fluorescence (*I*_D_) is recorded before (*I*_D before_) and after (*I*_D after_) bleaching of the acceptor and is described by the following equation:


$$\begin{array}{l} E_{FRET}=\;\;\;\frac{R_{0}^{6}}{R_{0}^{6\;\;\;}+\;\;\;R_{DA}^{6}} \end{array}$$

This method does not require expensive or complicated equipment, and does not need time-consuming training or extensive experience (see Table 1). Additionally, data acquisition is relatively fast compared with other FRET-based methods. Drawbacks of FRET-APB are the need for high laser powers during bleaching of the acceptor fluorophore, potentially leading to phototoxic effects, as well as low spatial resolution due to the necessary high acquisition speed and the analysis of only a small region of interest. Furthermore, filter sets and/or bandwidths should be carefully chosen to avoid possible crosstalk of donor and acceptor emission.

Another point to consider is that FRET-APB utilizes the intensity of the donor molecule fluorescence to calculate FRET efficiency and therefore is strongly affected by protein concentration. As a rule, a low donor/acceptor ratio will lead to increased FRET efficiencies whereas a high donor/acceptor ratio decreases FRET efficiency as the acceptor may be limiting. On the other hand, high expression levels of both proteins increase the possibility of the donor and acceptor fluorophore meeting by chance and could thereby artificially increase FRET efficiency. This phenomenon is known as bystander-FRET and should be taken into consideration for all FRET-based techniques. To avoid strongly differing POI concentrations in transient expression systems, both POIs can be expressed from a single T-DNA so that the preferable 1:1 ratio of donor and acceptor is achieved (Mehlhorn *et al.*, 2018; Denay *et al.*, 2019). Another point is, that depending on the cellular compartment and the mobility of the protein, the fluorescence of the acceptor could recover after the bleaching, even before an increase of the donor intensity can be detected. This effect might be enhanced by the inevitable delay between pre-bleach and post-bleach image acquisition. Therefore, highly mobile POIs and/or transient PPIs would be difficult to measure using APB. Another quantitative method to measure FRET overcoming some of the shortcomings of FRET-APB is FLIM, which is described next.

### Fluorescence lifetime imaging microscopy (FLIM)

Fluorescence lifetime (τ) is defined as the average time, usually in the nanosecond range, that a fluorophore remains in the excited state after excitation before returning to the ground state by emitting a photon. If two proteins interact, the fluorescence lifetime of the donor is decreased, and its fluorescence intensity is quenched. The fluorescence lifetime is an intrinsic characteristic for each fluorophore and therefore strongly differs between different fluorophores. FRET-FLIM is a non-intensity-based imaging method in contrast to the above-described FRET-APB, largely independent of protein concentration, making it particularly suitable for the quantitative analysis of PPIs in living cells, where fluorescence intensity can vary significantly. In order to measure the fluorescence lifetime of an FP in the so-called time domain, a pulsed laser source and special equipment for time-correlated single photon counting (TCSPC) is required: single photon-sensitive detectors and photon counting electronics (Becker *et al.*, 2004). The time between the laser pulse and emission of a single photon is measured for every individual photon and plotted as a histogram, which shows an exponential decay. From fitting this decay, the average fluorescence lifetime can be deduced (Biskup *et al.*, 2007; Weidtkamp-Peters and Stahl, 2017). The resulting FRET efficiency can be determined by recording the fluorescence lifetime τ of the POI labelled with the donor fluorophore in the absence (donor only sample) or presence of the putative interactor labelled with the acceptor fluorophore (FRET sample). The following equation describes the resulting FRET efficiency (*E*_FRET_, as a percentage) depending on the donor fluorescence lifetime in the presence (τ_DA_) or absence (τ_D_) of the acceptor:


$$\begin{array}{l} E_{FRET}=1-\frac{\tau_{DA}}{\tau_{D}}\times 100 \end{array}$$

One major advantage of FRET-FLIM is the quality of the acquired data. In contrast to other methods to investigate PPIs, FLIM data can also be used to determine quantitative data that can potentially show differences in the interaction strength or binding of different POIs at a high spatial resolution (see Table 1) (Weidtkamp-Peters and Stahl, 2017). Nevertheless, this requires some expert knowledge and advanced data analyses, as well as careful control experiments. Additionally, FLIM is largely independent of protein concentration. Therefore, FRET-FLIM measurements are widely considered as more reliable than the detection of FRET by APB and spectral imaging, and have been successfully applied *in planta* (Stahl *et al.*, 2013; Bücherl *et al.*, 2014; Somssich *et al.*, 2015; Long *et al.*, 2017; Weidtkamp-Peters and Stahl, 2017; Betegón-Putze *et al.*, 2021; Kaur *et al.*, 2021).

On the other hand, time domain FLIM data acquisition and processing are time-consuming and require a significant amount of training and experience. Furthermore, the necessary equipment, such as pulsed laser sources, TCSPC electronics, etc. are a quite expensive additions to a widefield or confocal microscope setup. An alternative that does not require cost-intensive TCSPC electronics and fitting of the data is the so-called frequency domain FLIM. Here, a continuous, modulated light source and a synchronized modulated detector are required to determine the phase-shifted fluorescent lifetimes of the donor fluorophore. Frequency domain FLIM measurements can be carried out at high speed, which is advantageous for monitoring dynamic processes. Nevertheless, time domain FLIM measurements show a higher precision even at low signal to noise ratios and can be used for more complex donors (Datta *et al.*, 2020).

A more general limitation of FRET-based methods, as in FRET-APB and FRET-FLIM, is the number of false-negative results resulting from inadequate photoselection of fluorophores (Bajar *et al.*, 2016). As mentioned, the dipole orientation (κ^2^) of the two fluorophores used for FRET should be (close to) parallel (Fig. 2). The more precisely the dipole orientations of the fluorophores are aligned in parallel, the more efficient the energy transfer from donor to acceptor and therefore the higher the FRET efficiency (reviewed in Weidtkamp-Peters *et al.*, 2022).

Another potentially problematic factor, apart from choosing the best possible FRET pair for the PPI experiment, is the position of the FP or enzyme at the N- or C-terminus of the POI. The protein class, the (subcellular) localization, and known functional domains can help to choose the optimal position of the reporter protein (Long *et al.*, 2018, 2020). In addition, a linker between the POI and the fluorophore could be used to improve FRET efficiency if this leads to an increased rotation of the fluorophore without increasing the distance between the two fluorophores, which could diminish FRET (Denay *et al.*, 2019).

Nevertheless, FRET-FLIM measurements are highly suited to validate PPIs in a quantitative way preserving spatio-temporal information of the POI. In addition, FRET-FLIM can also be used if PPI measurements of more than two POIs is necessary, as described below.

## PPI measurements of higher order complexes

### Combined BiFC and FRET

**Fig. 3.**
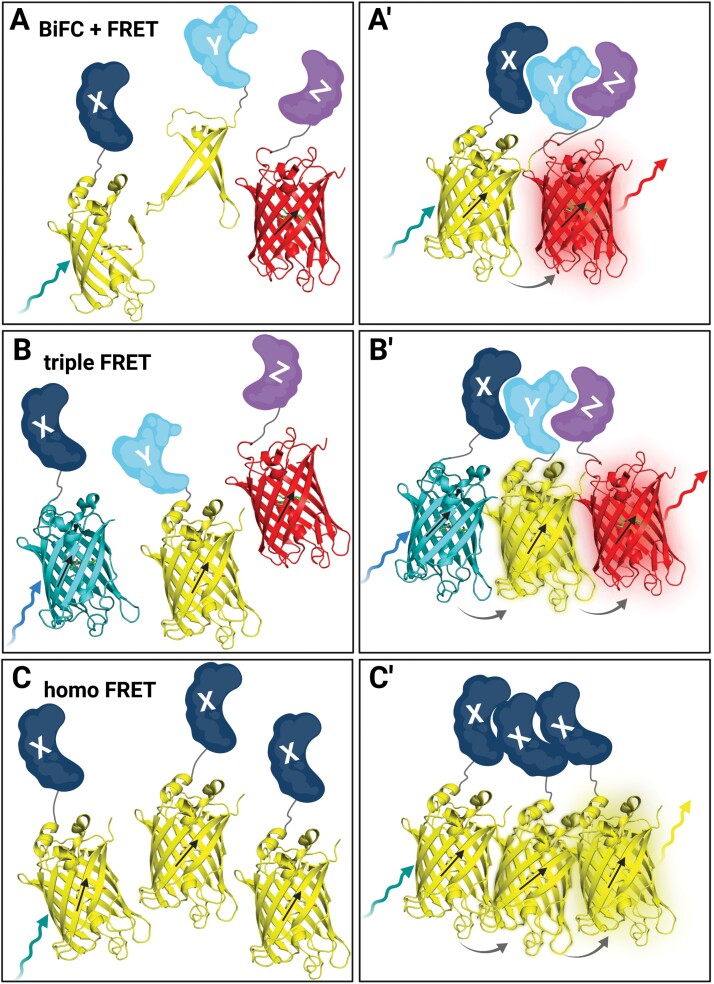
Techniques to measure PPI of more than two POIs. (A) In a combined BiFC–FRET experiment, two POIs (X, dark blue; Y, light blue) are fused to the N- or C-terminal part of a split FP (here, split 3D YFP structures in yellow), and a third POI (Z, purple) is fused to an acceptor FP (here, 3D mCherry structure in red). (Aʹ) In the case of interaction of all three POIs, the YFP parts are reconstituted and, after excitation (teal arrow), can transfer energy by FRET (grey arrow) to the acceptor FP (mCherry) which can emit light (red arrow). (B) In a triple FRET experiment, three POIs (X, dark blue; Y, light blue; Z, purple) are fused to three different FPs (here, 3D structures of CFP, cyan; YFP, yellow; and mCherry, red). (Bʹ) In the case of interaction of all three POIs, the three FPs come close enough to allow, upon excitation of CFP by blue light (blue arrow), energy transfer (grey arrow) by FRET to YFP as acceptor which then serves also as a donor and transfers energy via FRET (grey arrow) to mCherry which can then emit light (red arrow). (C) In a homo-FRET experiment, two or more of the same POI (X, dark blue) are labelled with an FP (here, 3D structure of YFP in yellow). (Cʹ) If the POI can form higher order complexes, energy can be transferred from one excited (teal arrow) FP to another, thereby depolarizing the emission of the FP (yellow arrow). Figure created with BioRender.com.

To identify and analyse putative homo- or heteromeric complex formation of more than two proteins, combinations of established methods to study PPIs are used. The combination of BiFC and FRET-FLIM offers the possibility to simultaneously test the interaction of three POIs while preserving high spatial resolution (Kwaaitaal *et al.*, 2010). Here, two POIs are fused to the N- or C-terminal part of the donor fluorophore, respectively. The third POI is fused to the acceptor fluorophore. Only in the case of complex formation of all three POIs can a reduction of the donor fluorescence lifetime or intensity be observed, for example by FRET-FLIM or FRET-APB measurements, respectively (Fig. 3B, Bʹ). An alternative approach without BiFC is described next.

### Triple FRET

Triple FRET, also referred to as three-colour FRET or two-step FRET, is another FRET-based method to simultaneously study interactions of more than two proteins, for example in higher order complexes consisting of different proteins. Here, the excited donor fluorophore (D) transfers energy to the first acceptor (A1), which at the same time serves as a donor for a second acceptor fluorophore (A2) (Horsey *et al.*, 2000). Thereby, energy can be transferred sequentially from D via A1 to A2, but also directly from D to A1 and from D to A2 (Fig. 3C, Cʹ). For the required energy transfer, the same prerequisites must be met that were described for conventional FRET above, but in this case for three FPs. Most importantly, the emission spectrum of D must overlap with the excitation spectrum of A1, and the emission spectrum of A1 must overlap with the excitation spectrum of A2. Before triple FRET-FLIM measurements can be carried out, a series of control experiments must be performed to ensure the measured FRET effect is caused by interaction and not by an artefact, for example quantification of fluorescence emission intensity of A1 and A2 with the excitation wavelength of D, to estimate spectral bleed through and crosstalk (Table 1).

One of the first attempts of triple FRET was used *in vitro* to investigate structural changes in DNA using sensitized emission as well as donor fluorescence and lifetime quenching as a read-out for FRET (Watrob *et al.*, 2003). The first experiments using triple FRET *in planta* in Arabidopsis mesophyll protoplasts also analysed sensitized acceptor emission and showed that 2-Cys peroxiredoxin forms decamers (Seidel *et al.*, 2010). Recently, the first attempts to establish three-colour FRET-FLIM *in planta* were performed (see Table 2) (Glöckner *et al.*, 2020, Preprint). Another technique to measure PPI of higher order complexes of the same POI is described next.

### Homo-FRET detection by fluorescence anisotropy measurements

FRET can also take place between members of the same kind of fluorophores because they also fulfil the requirements of FRET. This phenomenon of energy migration between identical fluorophores is called homo-FRET (Vogel *et al.*, 2009). The detection of homo-FRET can also be utilized to measure PPI between the same POIs, for example when they form dimeric or multimeric homomeric complexes. While homo-FRET does not impact fluorescence intensity or lifetime, it does affect the direction of the fluorescence emission of the examined fluorophores. This effect can be measured as fluorescence anisotropy *r* of a fluorophore (reviewed in Weidtkamp-Peters *et al.*, 2022). Here, a microscopic setup with a polarized light source (e.g. a laser) excites the fluorophores with a parallel dipole orientation, a process called photoselection. The steady-state fluorescence anisotropy *r* of the excited fluorophores can be measured by two detectors detecting the same emission bandwidth of the excited fluorophores but divided by a polarized beam splitter for detecting the intensity of the emitted light parallel (*I*_||_) and perpendicular (*I*_⊥_) to the polarized excitation light. Steady-state fluorescence anisotropy *r* can be described by the following equation:


$$\begin{array}{l} r=\;\frac{I_{∥}-I_{⊥}}{I_{∥}+2I_{⊥}} \end{array}$$

The fluorescence anisotropy *r* decreases in the case of PPI because of energy transfer to a fluorophore with the same properties in the vicinity of the next POI with a slightly different dipole orientation, thereby depolarizing the emitted fluorescence (Fig. 3C, Cʹ) (Weidtkamp-Peters *et al.*, 2022). Fluorescence anisotropy measurements can be combined with FRET-FLIM measurements to detect dynamic hetero- and homomeric complexes and their spatial distribution within a cell at the same time, which was shown *in planta* (Table 2) (Stahl *et al.*, 2013; Somssich *et al.*, 2015). Other spectroscopic methods described below can be used to detect PPI by correlating the fluctuations of the fluorescence of POIs as they move together.

### Fluorescence fluctuation microscopy to study PPI

Fluorescence correlation spectroscopy (FCS) is an advanced fluorescence technique that measures fluctuations in fluorescence of single molecules over time to quantify the concentration and diffusion coefficients of these molecules in a very small defined volume, such as in a confocal volume (Laursen *et al.*, 2016). This is achieved by focusing excitation light on to a sample (e.g. in a confocal or two-photon microscope), and the resulting fluctuation of fluorescence due to the movement or Brownian motion of the fluorescent molecules is statistically analysed. For FCS measurements, the number of fluorescent molecules must be relatively low (in the pico- to micromolar range) (Eigen and Rigler, 1994; Schwille *et al.*, 1999). Two POIs, if labelled with two spectrally distinct FPs, can be observed in two separate channels by fluorescence cross-correlation spectroscopy (FCCS). If the two proteins indeed interact, they will move together, as demonstrated with interacting receptor kinases in Arabidopsis roots (Table 2) (Wang *et al*., 2015). For FCS measurements, the same specialized microscopic equipment is needed as described above for FRET-FLIM measurements, because here too a very high temporal resolution of single photons is needed. These single molecule measurements are highly dependent on a very good signal to noise ratio which can be achieved by eliminating out-of-focus fluorescence as much as possible, such as by internal reflection microscopy, in particular variable angle total internal reflection microscopy (VA-TIRF) (Wang *et al*., 2015).

By using another FCS-based method called raster image correlation spectroscopy (RICS) which extracts fluorescence correlation from confocal image stacks combined with number and brightness analyses (N&B), two POIs labelled with spectrally different FPs can be analysed for their mobility, oligomeric state, and stoichiometry (including PPI) preserving spatio-temporal information, which has recently been successfully applied in Arabidopsis roots employing a conventional confocal microscopic setup (Table 2) (Clark *et al.*, 2016). The advantage of using RICS instead of FCCS is that no specific setup other than a conventional confocal microscope is needed (Table 1). Even though the analyses of the FCCS and RICS data is not as easy as, for example, in FRET-APB experiments, these techniques provide very valuable information on the mobility at the same time as on PPI.

### Knocksideways in plants (KSP)

Recently, an exciting new technique to measure and visualize PPI in transiently expressing *N. benthamiana* leaf epidermis was described which can be compared with an intracellular co-IP (Winkler *et al*., 2021). This technique combines the—in the animal field—well-established conditional ability of rapamycin to alter the localization of a bait protein and its interactors via the heterodimerization of FKBP and FRB domains. In KSP, this conditional heterodimerization is combined with rerouting interacting proteins to mitochondria upon rapamycin induction. The PPI of more than two POIs can thereby be directly visualized and quantified by FP-tagged POIs in conventional fluorescence microscopy (Table 2) (Winkler *et al*., 2021). So far, KSP has been used to improve the quality of interaction data acquired with split-ubiquitin, BiFC, and FRET approaches, as the addition of FKBP and FRB domains to these well-established methods can serve as an intrinsic positive control (Andersen *et al.*, 2016). It will be very interesting to see how applicable this new technique is in stable transgenic lines in the future, as KSP also offers, aside from PPI detection, a conditional compartmentalization and thereby a protein knockout tool.

One general consideration concerning all of the above-described PPI techniques that involve imaging is that the POI must be tagged or expressed in fusion with an FP or other protein (domain), and therefore the function of the POI might be impaired. Therefore, control experiments, such as rescue experiments using stable transformants in the respective loss-of-function mutants, should be carried out to test if the labelled POI is still functioning.

Nevertheless, the outstanding benefit of all described imaging-based PPI techniques is the preservation of spatio-temporal information of the involved POI and even quantitative data on the observed PPI (Table 1).

## Conclusions

In summary, many different techniques, most of them relying on the use of FPs, have been successfully applied in living plant cells, either in stably transformed plants or transiently in heterologous plant expression systems. Here, dynamic PPIs and complex formations can be investigated in a minimally invasive manner, whilst in most cases preserving the spatio-temporal characteristics of the POI. While we have summarized numerous pros and cons for each of the techniques to study PPI in this review, there is no one technique that fits all requirements. Which technique is best for a given research question depends on, for example, the expression system, POI abundance, and PPI strength and dynamics (Table 1). In the future, emerging techniques in the *in vivo* or correlative super-resolution microscopy field and/or in novel advances of data analyses could add more depth to the detection and/or quantification of PPIs. Here, novel, high-throughput techniques for improved visualization of PPI and the determination of dynamic *in vivo* binding affinities would help to decipher complex regulatory networks in plants. Additionally, the more insights into structural information on plant proteins become available, the more *in silico* predictions of PPI and even of PPI sites, such as in PlaPPISite, will become available (Ding and Kihara, 2019; Yang *et al.*, 2020), which can guide *in vivo* and *in planta* experiments. Due to the numbers of PPIs already predicted and/or verified in different plant species, computational networks of PPIs will become even more necessary to understand the complex and dynamic PPIs in a wider biological context.
